# A Path Forward: Promoting Microbial-Based Methods in the Control of Invasive Plant Species

**DOI:** 10.3390/plants10050943

**Published:** 2021-05-09

**Authors:** Maryam Shahrtash, Shawn P. Brown

**Affiliations:** Department of Biological Sciences, The University of Memphis, Memphis, TN 38152, USA; maryam.shahrtash@memphis.edu

**Keywords:** microbial consortia, invasive plants, integrated pest management, endophytes, disease facilitation

## Abstract

In this review, we discuss the unrealized potential of incorporating plant–microbe and microbe–microbe interactions into invasive plant management strategies. While the development of this as a viable strategy is in its infancy, we argue that incorporation of microbial components into management plans should be a priority and has great potential for diversifying sustainable control options. We advocate for increased research into microbial-mediated phytochemical production, microbial controls to reduce the competitiveness of invasive plants, microbial-mediated increases of herbicidal tolerance of native plants, and to facilitate increased pathogenicity of plant pathogens of invasive plants.

## 1. Introduction

Plants serve as habitats for microbes and microbial communities, which can colonize every plant tissue type [1,2,3]. These includes endophytes, which colonizes inter- and intracellular spaces within leaves, stems, and roots but are asymptomatic on the host [4], as well as epiphytes which colonize external surfaces of plant tissues. These microbes have varied direct and indirect interactions with plants that range from antagonistic (negative), mutualistic (positive) and everything in between [5]. In plant–microbe mutualisms, plants often release compounds that attract and feed the associated microbes, which may in turn secrete compounds that improve plant health and growth, thereby enhancing nutrient acquisition or making plants more resistant to environmental stressors. Prominent examples of mutualistic plant-microbe interactions are the symbioses between plants and nitrogen fixing bacteria and/or mycorrhizal fungi, that help plants grow in soils with low nutrient quality [6]. Conversely, antagonistic microorganisms negatively affect plant growth and/or health, which may be due to direct pathogenicity or a reduction in nutrient uptake rates. However, while we are beginning to better understand plant–microbiome interaction mechanisms [7], there is much about these interactions that remains unresolved [8]. With increased study of these interactions, experimental frameworks are beginning to emerge to investigate how microbiome manipulations can be best done to achieve management goals. 

To aid in the control of invasive plants, there has been increased interest in the development and utilization of microbial biocides as targeted direct biocontrol agents [9,10]. Several fungal biocides have shown promise in helping to control invasive plants, including *Fusarium oxysporum*, *Fusarium ploriferatum*, and *Trichoderma koningiopsis* which can partially control the invasive *Euphorbia heterophylla* (Mexican Fire Plant) [11], and *Albifimbria verrucaria* (formally *Myrothecium verrucaria* (Stachybotryaceae) [12]), which has been demonstrated to have biocidal action on numerous invasive plants including Kudzu [10], *Lygodium microphyllum* (old world climb fern) [13] as well as *Salvinia molesta* (floating fern) [14]. Additionally, the fungal genera *Colletotrichum*, *Phoma*, and *Sclerotinia*, as well as bacteria within in the genera *Xanthomonas* and *Pseudomonas* have also been demonstrated to have broad biocidal qualities [15]. However, complex multi-partite microbe–microbe interactions within plants can act in unforeseen ways to limit or modulate targeted goals. Plant-microbiome manipulative investigations are underexplored but have been suggested as a novel tool for invasive plant management [16]. Here, we argue that microbiome manipulations can be a powerful tool for helping to control invasive plants, but this emerging application has hitherto been underutilized and poorly studied. 

Given the potential importance of microbial based invasive plant management, we examine existing research on the interactions between invasive plants and their microbiome and posit the impacts of manipulations of these microbial communities to favor invasive plant suppression and control. Together, these studies reflect the need for additional investigations and a broader scope of research into the applications of invasive plant–microbiome and microbe–microbe interactions as a microbial-based management tool. Microbial-informed invasive plant control strategies could include the introduction of plant pathogenic microbes or microbial inhibition of beneficial plant-associated microbes; together, these will act to reduce invasive plant fitness and ecological impacts. Application of synthetic and/or naturally isolated microbial communities or consortia composed of multiple species with different modes of action and various microbe–microbe interactions could be an alternative and complimentary approach in invasive plant management [17].

While biocontrol is still and will likely remain an integral part of invasive plant management, studies into microbiome manipulations to suppress invasive plants are needed to improve efficacy of biocontrol agents as well as provide control opportunities where biocontrol or herbicidal applications are prohibited or otherwise problematic. 

**Figure 1:**
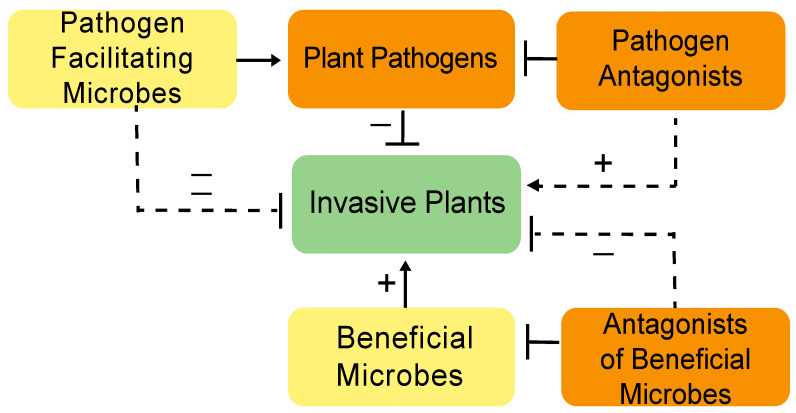
Diagrammatic representation of potential microbial interactions with invasive plants. Direct (solid lines) and indirect (dashed lines) impacts (positive [+] dignified with arrows and negative [−] signified with capped lines) on invasive plant fitness are indicated.

Classical biocontrol applications can carry risks; one of the major challenges with a classic biocontrol approach is the adaptation and spread of resistant plant genotypes, which will provide diminishing returns over time [18]. Another drawback is often the lack of biocontrol host specificity [19]. Many plant pathogens used for biocontrol can infect alternative hosts leading to unintended mortality of non-invasive and/or non-target plants. A potentially fruitful frontier in plant management could be the development of microbial consortia that negatively impacts invasive plant fitness through either decreasing their tolerance to biotic or abiotic environmental stress or by improving native plant competitiveness in invaded areas. Microbial consortia are likely to be more effective than individual microbial species introductions as communities tend to be more robust to environmental fluctuations. Also, the probability of plants developing systemic immune responses to consortia is lower than that of individual taxa. In microbial consortia, each member within the consortia interacts and impacts either directly or indirectly with the plant host and/or with one another which ultimately creates an interactive network that impacts host plant fitness and health [20]. We illustrate how these interactive networks may influence invasive plants to achieve control goals (Figure 1). Consortia can be governed by the presence of keystone species (hubs), the major determinants of the microbiome network structure [21], or it may involve tripartite or multipartite interactions [22,23]. Investigations into appropriate network structure for each target invasive plant are needed to develop individual control strategies.

This complex interactive relationship can occur via metabolite or hormone exchange, or through signal transduction pathways [24,25]. Microbial consortia can exhibit complex functionality and their robustness to environmental fluctuations needs to be extensively examined before applications are developed for environmental use. There is an expanding body of literature showing that plant secondary metabolites can alter plant microbiomes and result in differential microbial community assembly [26,27]. Plants release a large proportion of their photosynthates through the soil rhizosphere [28,29] which activates nutrient mobilizing symbionts and/or beneficial plant growth-promoting (PGP) bacteria [30,31]. Plant secondary metabolites impact microbiome structure by acting as signaling molecules, nutrients sources, or as direct toxins [27,32]. Some studies have demonstrated that invasive plants can produce more secondary metabolites than native plants [33,34]. These secondary metabolites facilitate nutrient cycling [35] which may allow invasive plants to outcompete native species. For example, benzoxazinoid indole-derived compounds can function as allelochemicals or protectants against pathogens [36] and act as chemoattractant for (PGP) bacteria in the rhizosphere [37] in invasive plants. Additionally, plant growth promoting rhizobia have been demonstrated to increase scavenged nutrient translocation into legumes, with phosphate additions driving increase nodulation production to facilitate plant growth [38,39]. This, in addition to rhizobia-mediated reduction of ethylene stress associated with degradation of the ethylene precursor molecule 1-aminocyclopropane-1-carboxylic acid (ACC) by ACC deaminase (ACCd) can lead to plant stress reduction [40,41], which facilitates increased resistance to phytopathogens via indirect and direct actions [42]. Together, the utility of considering the integration of plant–microbe and microbe–microbe interactions to alleviate pathogenicity of native plants in the face of biocidal control of proximate invasive plants becomes clear.

Here, we discuss several scenarios that have been envisaged whereby modification of invasive plant or native plant microbiomes can be considered as a promising sustainable approach in invasive plant control and recovery by native plants. We encourage the research community to incorporate multipartite microbial interactions into the development of the next generation of invasive plant management strategies. These include improving beneficial native plant phytochemical production, reducing the competitiveness of invasive plants, increasing herbicidal tolerance of native plants, and facilitating increased pathogenicity within invasive plants.

## 2. Research Directions

### 2.1. Improving Beneficial Native Plant Phytochemical Production

One of the most promising directions of microbial-mediated invasive plant management is perhaps the least well studied. Factors allowing for the success and establishment of invasive species in non-native ranges have been investigated for a long time [43]. According to the novel weapons hypothesis, allelopathic chemicals released by invasive species more effectively inhibit plants from outside of that species’ native community than do those of its native range [44]. One major reason is the more ‘successful’ exotic plants have diverse plant secondary metabolites which may protect against biotic and abiotic stress [45]. These allelopathic effects can accelerate plant invasions [46,47]. Conversely, if the allelopathic potential of native species could be maximized in similar ways, it may provide additional protection against exotic invasions. The allelopathic interactions between native and invasive plants are poorly studied but some studies suggest that allelochemical production by native plants can reduce invasive plants growth [48]. For instance, *Pinus ponderosa* was reported to allelopathically suppress the growth of *Centaurea stoebe*, a noxious weed in the western USA [49]. Further, the presence of pine litter alters soil chemical dynamics, thereby altering the composition soil microbes which can, in turn, further suppress *Centaurea* [50]. 

More detailed elucidation of plant–plant–microbe interaction mechanisms is needed to fully integrate these interactions into management and restoration strategies, but these interactions are promising in theory. It was reported that the European invasive plant, *Alliaria petiolate* can limit native plants’ mycorrhizal fungal richness and colonization rates through releasing secondary metabolites, which in turn negatively impacts native plant health and fitness [51,52]. To provide native plants with a competitive advantage, emphasis needs to be directed toward identifying, selecting and harnessing microbial communities that can improve and/or maximize the native plants’ secondary metabolites or allelochemicals production. This could take the shape of exogenous application of selected microbial consortia or as seed coatings used in restoration planting. Unraveling the mechanisms through which microbes control the production of secondary metabolites like allelochemicals and vice versa will help us to pursue the development of management strategies that imitate the structure and function of native plant ecosystems while reducing chemical inputs on the environment. We view this as an important but understudied potential management tool and one that desperately needs additional research to harness this potential.

### 2.2. Reducing Competitiveness in Invasive Plants

Previous studies have found that exotic and/or invasive plants tend to interact differently or more favorably with microbes outside of their native range [53] and can physically alter microbial network structure in invaded ranges [54]. This suggests that soil microbes could be a key component to invasive plant establishment and continued fitness in invaded areas. Many studies reveal that invasive plant microbiomes play a large role in their ability to survive in adverse environmental conditions through mitigation or alleviation of environmental stressors [55]. Understanding the role soil- and plant-associated microbes play in the invasion process will help find strategies to reduce plant fitness in invaded ecosystems. Invasive plants have a competitive advantage over native species [56] and usually have higher net primary productivity (NPP) and greater nitrogen scavenging ability than native plants [57,58]. The rapid radiation of invasive plants can be partially accounted for by co-introduction of pathogens or shifts in abiotic conditions in introduced ranges [46,59], increased abundance and activity of symbiotic microbes [60,61], and higher mineralization rates of nitrogen [62,63], which can be directly influenced by plant endophytes [64]. Soil communities can also be altered following the introduction of invasive species [54], which can account for the higher nitrification rates, a phenomenon that shifts competitive outcomes in favor of invaders and against natives [60]. Invasive plant-mediated shifts in soil properties can further exacerbate microbial community alterations, which can further favor establishment [65]. 

Manipulating microbial communities within and among invasive plants through the introduction of new microbial populations or providing favorable conditions for shifting established population ratios is a largely uninvestigated option to reduce the competitiveness of invasive plants. This can lead to shifting competitive probabilities in favor of native species, allowing favorable interspecific competition outcomes for native plants. However, it should be noted that utilization of such microbial inoculates, even if favorable outcomes can be achieved, is not without controversy [66], but we need to balance the net benefit with potential ecosystem harm when making these decisions. One avenue towards microbial-mediated invasive plant management is utilizing microbial consortia that can indirectly suppresses invasive plant growth, but development and validation of consortia prior to environmental testing can be difficult [67]. Suppression of invasive plants can occur through inhibition of microbes that mostly benefit the invasive plant, which increases invasive plant fitness. Inhibiting these beneficial microbes will result in net reduction in invasive plant fitness as nutrient acquisition (among other potential mechanisms) capability will be reduced. Evidence for the efficacy of such an approach comes from the counter example of utilizing endophytes and other microbes to increase plant productivity by means of pathogen alleviation [68], whereby endophytes can reduce pathogenicity, thereby benefiting the plant. This other side of the coin is an obvious extension, but one that has been relatively unexplored. Another potential approach is to alter the native plant community near invasive plants to facilitate subsequent cascading effects on soil microbial communities [69]. For instance, planting cover crops in infested areas could affect the quantity and quality of root exudates to the soil, which may in turn affect soil biogeochemical processes and nutrient pools and change the microbial community in invaded areas that favor native plants, although, unfortunately, this type of next-order manipulation is not a major area of active research [70]. 

### 2.3. Increasing Herbicide Tolerance in Native Plants

If native or otherwise desirable plants in close proximity to invasive plants can be made more resistant to common herbicides, or less responsive to herbicidal drift, then direct herbicidal application to control invasive plants will produce less ancillary damage. If plant antioxidant content and reactive oxygen species (ROS) scavenging capability could be increased in native plants, they might better tolerate many herbicidal actions. Herbicides can trigger ROS generation in microbes [71] and these ROSs can increase plant cellular damage [72]. It has been shown that certain microbes can function as bio-remediators and convert organic pollutants and xenobiotics into nontoxic products and utilize them a source of carbon, phosphorus, sulfur or nitrogen [73]. Several reports have implicated the significant roles of microbes in degrading the active ingredients of some herbicides [74]. For example, Atrazine can be metabolized by some rhizospheric bacteria including *Arthrobacter* sp. [75], *Pseudomonas aeruginosa*, and *Clavibacter michiganense* [76]. Some *Pseudomonas* strains can metabolize atrazine into cyanuric acid which is then hydrolytically changed to ammonia and carbon dioxide [76]. Manipulating native plant microbiomes in favor of these herbicidal degraders may provide a level of protection to the native plant, but this protective ability is likely to be context-dependent based on the herbicidal mode of action and half-life in soil. Here, we present an incomplete list of taxa that have documented herbicide degradation capabilities. While this is only intended to provide a snapshot of how some microbes can degrade or otherwise transform herbicides, this can serve as a list of potential microbial targets that may have utilization potential for protection against herbicidal action and should be investigated further (Table 1).

An additional mechanism to confer tolerance to herbicides in native plants could be priming tolerance through microbial-based induction of ROS scavengers within native plants [89,90] or induction of jasmonic acid, oxylipins and salicylic acid production, leading to induction of tolerance responses to herbicide oxidative stress [90]. Another ecologically sound approach to boost native plant tolerance to oxidative stress from herbicides is inoculation with plant growth promoting microbes (PGPM) and certain mycorrhizal fungi. PGPMs can enhance plant growth and resistance to stressors through a wide variety of mechanisms including regulating plant hormones and other phytochemicals, improving nutrition acquisition, siderophore production, enhancing the antioxidant system and activation of induced systemic resistance (ISR) [91]. Most commercially available biofertilizers contain single species inoculants that promote plant growth; however, consortia inoculation might provide higher growth promotion and stronger disease resistance due to cumulative synergistic effects of consortia inoculation over individual inoculations [70].

As discussed earlier, some herbicides cause oxidative damage in plants. For example, glyphosate inhibits the shikimic acid pathway and consequently the production of ROS in tissues [92]. Another mechanism to protect native plants is through the upregulation of phenylpropanoid pathways and boosting the antioxidant system through exogenous treatment of native plants with phytohormones or microbial partners that causes upregulation within the plant [93]. More research into microbial-mediated upregulation of protective pathways needs to be conducted.

### 2.4. Facilitating Increased Pathogenicity in Invasive Plants

Numerous studies indicate that plant-microbe interactions can improve plant tolerance to biotic stress and/or alleviate pathogenicity effects via multiple mechanisms including secretion of antimicrobial compounds [42,94,95,96], hyperparasitism [97], and competition for resources such as nutrients or space [98]. However, most research on direct interactions between microbes and pathogens focuses on pathogen mitigation and symptom alleviation [8,99]. Investigations into microbial-mediated pathogen facilitation and increased pathogenicity have not been extensively studied but may have enormous potential to suppress invasive plants [16]. Some plant-associated microbes produce metabolites that can promote pathogen development and facilitate disease [100]. Further, pathogens might exploit specific plant microbes to enhance their pathogenicity or plant susceptibility, and this connection might be driven by production of a plethora of secondary metabolites or hormones by endophytes [99] which may directly or indirectly (via inhibition of a mycoparasite, for instance) facilitate pathogenicity. By developing a framework whereby microbiome manipulations can increase a pathogen’s efficacy, invasive plants can be dramatically suppressed via naturally occurring environmental pathogens. Facilitation occurs when one microorganism enhances the development or growth of another. This facilitation may also be due to ecological interactions including competitive exclusion or niche partitioning [101]. By investigating and understanding the dynamics of the invasive plant micro- and mycobiomes, we can develop strategies for modification and manipulation of these communities [16] to favor successful colonization and growth of taxa that facilitate pathogen virulence, or taxa that negatively impact invasive PGPMs, thus resulting in suppression in invasive plants and a reduction in plant fitness. This is an emerging field of study, but one that we feel will be of increasing importance with a growing emphasis on sustainable and non-chemical controls of invasive plants [15]. Targeting invasive plant microbiomes is a novel method of integrated management of invasive plants that deserves to be explored. Identifying, understanding, and the utilization of microorganisms or microbial products to reduce invasive plant fitness are becoming more central parts of sustainable agriculture. To better understand the potential for microbial-mediated facilitation of pathogenicity of invasive plants, and to stimulate the research community into action, it is useful to briefly examine some mechanisms in induction of signaling cascades in plants by microbes.

**Figure 2:**
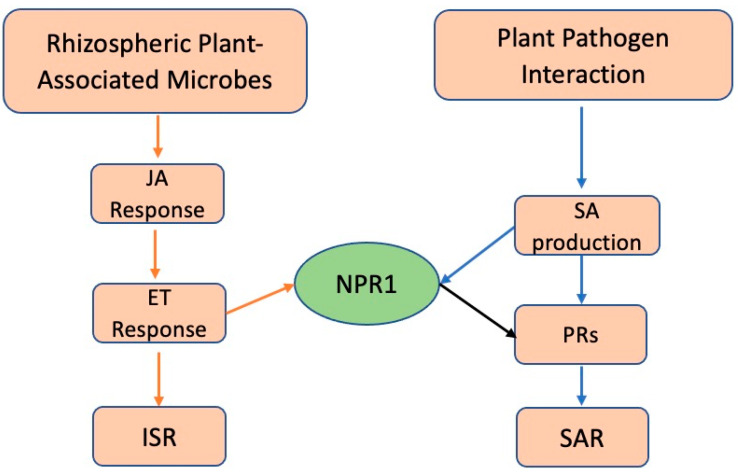
Schematic representation of the common signal-transduction pathways leading to pathogen-induced systemic acquired resistance (SAR) and rhizosphere-mediated induced systemic resistance (ISR) inspired by [114] for *Arabidopsis thaliana* but applicable to plants in general. Crosstalk between the two pathways occurs through the activation of NONEXPRESSOR OF PATHOGENESIS-RELATED GENES1 (NPR1). Non-pathogenic plant-associated microbes, usually from the rhizosphere, can trigger the SAR pathway as well as ISR. In the rhizosphere-mediated ISR pathway, components from the jasmonic acid (JA) and ethylene (ET) responses act in sequence to activate a systemic resistance response (orange arrows). Pathogenic agents could activate the pathogen-induced SAR, through the activation of NPR1 (blue arrows), leading to the expression of PATHOGENESIS-RELATED genes (PRs) (black arrow). NPR1 also mediates crosstalk between the SA signaling pathway.

Plant responses to colonization by microbes can be broadly categorized into one of two main categories SAR (systemic acquired resistance), triggered by plant pathogens, and ISR (induced systemic resistance), triggered by root-colonizing mutualistic microbes (Figure 2) [102,103]. Although both pathways share many common signaling components, their elicitors and regulators are distinct. The conserved microbe-specific elicitors, referred to as microbe- or pathogen-associated molecular patterns (MAMPs or PAMPs), are perceived by the plants’ innate immune systems pattern recognition receptors (PRRs). Examples of elicitors include flagellin (Flg), elongation factor Tu (EF-Tu), peptidoglycan (PGN), lipopolysaccharides (LPS), Ax21 (activator of XA21-mediated immunity in rice), fungal chitin, and β-glucans from oomycetes, among others, can be recognized by plant surface localized PRRs [104]. MAMP elicitors, upon perception, can trigger a SAR signaling cascade which is characterized by increased levels of the hormone salicylic acid (SA) which, activates the expression of a large set of pathogenesis-related (PR) genes through the induction of the redox-regulated protein NON-EXPRESSOR OF PR GENES1 (NPR1) leading to the activation of the defense responses [105,106]. The mechanisms underpinning NPR1 action have been well documented [107] and it plays a major role in direct pathogenicity and defense in plants. In SAR, MAMPs elicitors activate ISR signaling pathway which is mediated by an SA-independent pathway where Jasmonic Acid (JA) and ethylene (ET) play major roles, and typically functions without PR gene activation [105,106]. Some studies have suggested that NPR1 may also be required for the ISR triggered by certain rhizospheric microbes [106,108]. Some studies also suggest that ISR is required for SA accumulation in plants [109,110,111]. ISR eliciting rhizospheric microbes activate plant defense responses which are often effective against a broad spectrum of plant pathogens [112] which could be an avenue to increase native plants fitness. However, there are cases in which harmless or even beneficial microbes can assist pathogen establishment [113], which demonstrates the importance of additional research into these interactions before microbial-mediated disease facilitation and/or protection can be fully developed.

### 2.5. Concerns and Potential Problems with Microbial Deployment 

To establish a successful microbial-mediated control program, there are many potential concerns that must be taken into account. Introduction of microbes into nature, even if these microbes are already occurring in a particular environment, can carry risk. There is always a risk of non-target associations with applied microbes, which may be problematic particularly for pathogenic applications [115]; extensive host-specificity testing procedures are needed to predict the potential non-target effects. Additionally, development and implementation of microbial applications must be approached from a risk assessment framework [116]. Some have argued that the risk associated with potential unforeseen consequences of microbial inoculants is unacceptable [66] as well as being confronted by too many potential ethical and legal issues [117]. This wariness is understandable, and is largely justified by highlighting the complexity of these systems [118,119] which may occlude potential problems until too late. However, one could argue that the economic and ecological cost of doing nothing [120,121,122] is far greater than a calculated risk, as long as controlled in planta validations and detailed cost–benefit analyses have been conducted [123]. Extensive work is must be done to study pathogenicity, adaptability, colonization, reproduction, dispersal, and survival efficiency of any potential microbial agents used for biocontrol [124], but this is a desperately needed area of additional research in the future.

## 3. Research Gaps, Future Directions, and Conclusions

Several studies have explored the role of particular microbes for biocontrol or plant protection services. However, there have only been limited investigations into field-scale investigations and utilization of a consortia approach to either control invasive plants, or benefit native plants to shift competitive outcomes when threatened with invasive plants [79,125,126,127,128]. Here, we advocate for additional research to advance sustainability and an integrated microbiological approach to help suppress invasive plant fitness as a potential additional tool for land managers. We identify three main, but not complete, research priorities that need to be investigated to move this field out of its infancy: (1) the development of integrated predictive models to understand the multipartite effects of pathogen–endophyte interactions associated with invasive plants; (2) the definition and elucidation of core pathogen–endophyte combinations on invasive plants to develop targets for additional investigations; (3) the elucidation of how microbial consortia mechanistically interact with hosts, environments, and management strategies in order to develop targeted application plans.

Invasive plant species are one of the challenges facing the world, leading to great economic losses. Inclusion of microbial-based management options for invasive plant management should be investigated with the goal of ultimately reducing invasive plant fitness. Combinations of individual microbes with complementary or synergistic traits may increase the competitive ability of native plants and/or susceptibility of invasive plants which may ultimately reduce invasive plant fitness in the invaded range. Not only does the effectiveness of individual microbes need to be examined for invasive plant control, but so does the interrelation, strength, and directionality of interactions between taxa. These strategies should be incorporated in invasive plant management programs. Here, we implore the invasive plant management research community to incorporate microbial dynamics into explorations of control strategies. The control potential of such methods is promising, but additional investigations are needed to move these strategies into active development.

## Figures and Tables

**Table 1:** List of bacterial (top) and fungal (bottom) taxa that have demonstrated herbicidal biodegradation or mineralization capabilities. Presented are species/strain names, herbicides and mode of actions of degradation.

Species/Strain	Herbicide	Mode of Action	Citation
**Bacteria**			
*Pseudomonas* sp. ADP.	Atrazine	Mineralization	[77]
*Burkholderia* (*Pseudomonas) cepacia* DBO1(pRO101)	2,4-Dichlorophenoxyacetic acid	Biodegradation	[78]
*Comamonas* sp. SWP-3	Swep	Hydrolysis	[79]
*Alicycliphilus* sp. PH-34	Swep	Hydrolysis	[79]
*Sphingomonas wittichii* DC-6	Chlorocetanilide	Mineralization	[80]
*Pseudomonas syringae*	Triazole	Biotransformation	[81]
*Xanthomonas citri*	Triazole	Biotransformation	[81]
*Enterobacter cloacae* K7	Glyphosate	Biodegradation	[82]
*Arthrobacter* sp. GLP-1	Glyphosate	Biodegradation	[82]
**Fungi**			
*Trichoderma viride*	Pirimicarb	Biodegradation	[83]
*Trichoderma harzianum*	Pirimicarb	Biodegradation	[83]
*Nocardioides* sp. MFC-A	Mefenacet	Hydrolysis	[84]
*Rhodococcus rhodochrous* MFC-B	Mefenacet	Hydrolysis	[84]
*Stenotrophomonas* sp.	Mefenacet	Hydrolysis	[84]
*Polyporus tricholoma*	Paraquat	Enzymatic Degradation	[85]
*Cilindrobasidium leave*	Paraquat	Enzymatic Degradation	[85]
*Deconica citrospora*	Paraquat	Enzymatic Degradation	[85]
*Aspergillus terrus*	Triazole	Biotransformation	[81]
*Penicillium chrysogenum*	Triazole	Biotransformation	[81]
*Mortierella* sp. strain Gr4	Isoproturon	Hydrolysis	[86]
*Phoma* cf. *eupyrena* Gr61	Isoproturon	Hydrolysis	[86]
*Alternaria* sp. strain Gr174	Isoproturon	Hydrolysis	[86]
*Plectosphaerella cucumerina* AR1	Nicosulfuron	Hydrolysis	[87]
*Phanerochaete chyrosporium*	Atrazine	Biotransformation	[88]

## Data Availability

No new data were created or analyzed in this study. Data sharing is not applicable to this article.
